# Determinants of adherence to heart failure medication: a systematic literature review

**DOI:** 10.1007/s10741-012-9321-3

**Published:** 2012-06-22

**Authors:** R. Oosterom-Calo, A. J. van Ballegooijen, C. B. Terwee, S. J. te Velde, I. A. Brouwer, T. Jaarsma, J. Brug

**Affiliations:** 1 Philips Research, Eindhoven, The Netherlands; 2 EMGO Institute for Health and Care Research and the Department of Epidemiology and Biostatistics, VU University Medical Center, Amsterdam, The Netherlands; 3 EMGO Institute for Health and Care Research and the Department of Health Sciences, Faculty of Earth and Life Sciences, VU University Amsterdam, Amsterdam, The Netherlands; 4 Department of Social and Welfare Studies (HAV), Linköping University, Linköping, Sweden

**Keywords:** Determinants, Medication adherence, Heart failure

## Abstract

A systematic literature review was conducted to summarize the existing evidence on presumed determinants of heart failure (HF) medication adherence. The aim was to assess the evidence and provide directions for future medication adherence interventions for HF patients. Based on a search in relevant databases and a quality assessment, eleven articles were included in the review. A best evidence synthesis was used to combine the results of presumed determinants that were found more than once in the literature. Results were classified according the World Health Organization’s (WHO) multidimensional adherence model. Results demonstrated a relationship between having been institutionalized in the past (including hospitalizations and nursing home visits) and higher adherence levels. This finding is related to the healthcare system dimension of the WHO model. The presumed determinants related to the other dimensions, such as social and economic factors, condition-related, therapy-related, and patient-related factors of the multidimensional adherence model all had inconsistent evidence. However, there was also an indication that patients’ educational level and the number of healthcare professionals they have visited are not related to higher adherence levels. Based on the current review, HF patients who have been institutionalized in the past are more adherent to HF medication. Many other presumed determinants were investigated, but displayed inconsistent evidence. Due to the lack of evidence, it was not possible to make recommendations for future interventions.

## Introduction

Heart failure (HF) is a chronic cardiac condition prevalent especially among the elderly, characterized by high mortality and hospitalization rates [1]. The European Society of Cardiology [2], American College of Cardiology/American Heart Association [3], and Heart Failure Society of America [4] guidelines for HF treatment specify both pharmacological and non-pharmacological treatment strategies. The objectives of pharmacological treatment in HF include reduction in mortality and morbidity and prevention of further worsening of the condition [2].

Adherence to medication is defined as the extent to which patients take medications they have been prescribed [5]. Non-adherence to medication is pervasive among patients of chronic diseases, although there is no standard as to what constitutes adequate adherence [5]. A meta-analysis of 569 studies on patient adherence to medication reveals that the rate of non-adherence is on average 24.8 % in the general patient population [6]. Among HF patients, the rates of adherence reported in studies vary between 10 and 98 %, depending on the measurement instruments used [7]. Non-adherence to HF medications is related to poor clinical outcomes and high healthcare costs [8].

Adherence to medication can be promoted through various interventions. A recent study that measured healthcare professionals’ strategies to promote medication adherence showed that educational/cognitive interventions were the most common, followed by counseling/behavioral interventions [9]. In order to devise effective tailored and targeted interventions, it is important to determine which factors are associated with, and may reduce levels of, non-adherence. The World Health Organization (WHO) defines five dimensions of adherence [10]. These are social and economic factors, healthcare system-related factors, condition-related factors, therapy-related factors, and patient-related factors.

A best evidence synthesis is a method of synthesizing evidence used in literature reviews, in which the best available evidence is utilized to produce and defend conclusions [11]. A possible conclusion to be reached as a result of performing best evidence synthesis may be that the available evidence does not allow reaching any conclusions. Performing this method of best evidence synthesis includes assessing the internal and external validity of studies and weighing the evidence based on the studies’ scientific rigor. Before performing the best evidence synthesis, criteria for rating the levels of evidence should be defined. These can be derived from previous literature [11]. In order to summarize the available evidence and reach conclusions about the levels of evidence, we relied on the principles of a best evidence synthesis.

The aim of the current systematic literature review is to assess the level of evidence for presumed determinants of medication adherence and make recommendations for future interventions to increase adherence levels. This is the first review to systematically assess the evidence for determinants of HF medication adherence, using a methodological quality assessment [12] and a best evidence synthesis [11, 13, 14].

## Method

The study selection process included four steps: a search in the electronic databases, scanning of titles and abstracts to select relevant articles, scanning the full text articles and applying inclusion and exclusion criteria to select eligible articles, and conducting a methodological quality assessment to remove poor-quality studies.

### Search of literature in electronic databases

**Fig. 1:**
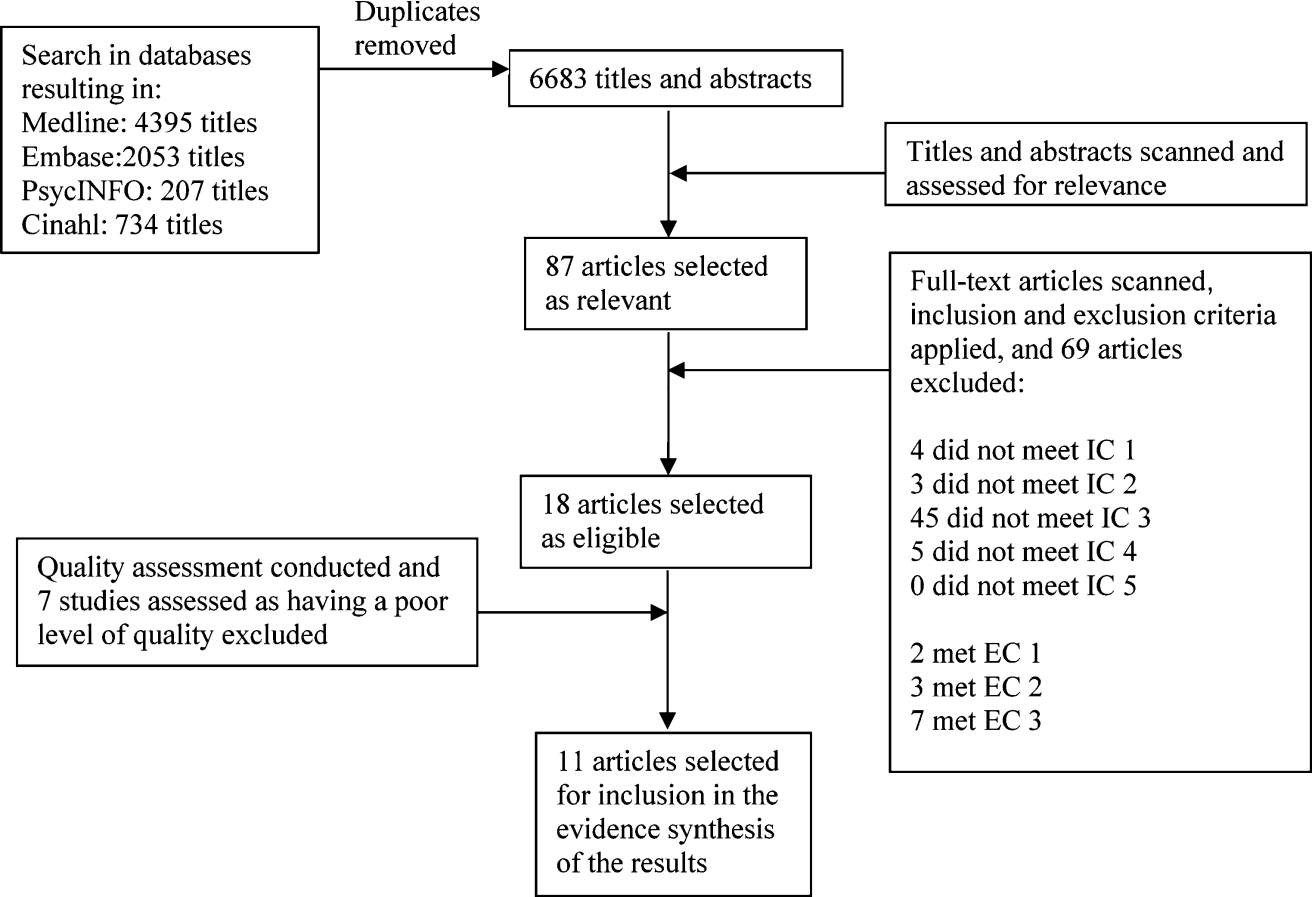
Flowchart of article selection process

The literature search was conducted in five electronic databases (Fig. 1) in August 2010. Limits were set on full text but not on dates. The search included titles on HF behaviors associated with both pharmacological (medication adherence) and non-pharmacological (lifestyle) recommendations. The results on the HF self-care behaviors other than medication adherence have been reported elsewhere [15].

### Review of titles, abstracts, and full text articles

In the second step of the article selection process, two authors (ROC and AJvB) independently scanned the titles and abstracts generated by the search. The two authors then made decisions about inclusion based on the relevance of the articles to the topic of the review, compared their decisions and reached consensus. If there was lack of consensus, a third author was consulted (CBT).

Next, an inclusion and exclusion criteria list was devised, so that it can be used to select eligible studies in the next step of the study selection process. Studies were selected if they met the following inclusion criteria (IC):

1. At least 50 % of the sample consisted of HF patients.
2. One or more presumed determinants of medication adherence were investigated
3. Medication adherence was (one of the) main outcomes.
4. Quantitative results were reported.
5. Published in English.

Studies were excluded if they met the following exclusion criteria (EC):

1. Review papers.
2. Evaluations of interventions were their main purpose.
3. Descriptive studies.

In the third step of the article selection process, the two authors scanned the full texts of the selected articles, and selected articles for inclusion based on the criteria independently and compared their selections. If there was lack of consensus on methodological issues, a third author was consulted (CBT). If there was disagreement about clinical aspects, a fourth author was consulted (TJ).

### Methodological quality assessment

A checklist that was devised (Table 1) based on the Quality in Prognosis studies (QUIPS) tool, designed for systematic reviews of prognostic studies through international expert consensus [12]. The QUIPS tool contains six categories assessing (1) bias due to patient selection, (2) attrition, (3) measurement of prognostic factors, (4) outcome measurement, (5) confounding on statistical analysis, and (6) confounding on presentation. To strengthen the discriminative capacity of the checklist, the description of each category was transformed into a set of individual questions that were scored separately.

**Table 1:** Checklist of quality criteria used in the quality assessment

Methodological issue	Questions addressed	Scoring
Theoretical background	1. Is a theoretical background presented, to which the motivation for conducting the study and/or the hypotheses are linked?	Y = 3, NR = 2, N = 1
Study participation	2. Is the study population clearly described in terms of age, gender, and important HF characteristics?	Y = 3, NR = 2, N = 1 Y = 3, NR = 1, N = 2
	3. Is the percentage of eligible subjects who participated in the study (response rate) adequate?	
Sampling	4. Are patients who participated in the study similar to eligible non-participants, in terms of age, gender, and important disease characteristics?	Y = 3, NR = 1, N = 2
Study attrition	5. Is the percentage of subjects available for analysis adequate (i.e., >70 %)?	Y = 3, NR = 1, N = 2 Y = 3, NR = 1, N = 2
	6. Were reasons for loss to follow-up presented and assessed during the study for possible systematic attrition?	
Determinant/correlate(s) measurement	7. Are clear definitions of each determinant and/or correlate provided?	Y = 3, NR = 2, N = 1 Y = 3, NR = 2, N = 1 Y = 3, NR = 1, N = 2 Y = 3, NR = 2, N = 1
	8. Are clear operationalizations of each determinant and/or correlate provided?	
	9. Are the measurement instruments used for the measurement of the determinants and correlates reliable and valid?	
	10. Were the measurement approach, time and place of measurement of the determinants and/or correlates standardized or conducted in a way that limits systematically different measurement?	
Outcome variable(s) measurement	11. Are clear definitions of each outcome variable provided?	Y = 3, NR = 2, N = 1 Y = 3, NR = 2, N = 1
	12. Are clear operationalizations of each outcome variable provided?	
	13. Are the measurement instruments used for the measurement of the outcome variable(s) reliable and valid?	Y = 3, NR = 2, N = 1
	14. Were the measurement approach, time and place of measurement of the outcome variable(s) standardized or conducted in a way that limits systematically different measurement?	Y = 3, NR = 2, N = 1
Statistical analyses	15. Is the percentage of missing values adequate (i.e., <30 %)?	Y = 3, NR = 1, N = 2
	16. Were multivariable analyses performed? If yes, was it clearly described which variables were included in the (multivariable) model(s)?	Y = 3, NR = 1, N = 2
General question	17. Were there any other important flaws in the design or analyses of the study?	Y = 3, NR = 2, N = 1

*Y* yes, *N* no, *NR* not reported

In the fourth step of the article selection process*,* the two authors independently assessed the quality of each study and compared their results. Specifically, a quality score of between 1 and 3 was provided for all items on the quality checklist (Table 1), for each study separately. If there was lack of consensus, a third author was consulted (CBT). After consensus was reached, an average quality score (range 1–3) was computed for each study. Studies that received an average quality score of between 2.5 and 3.0 were regarded as good-quality studies, those that received an average score between 2.0 and 2.4 or less were regarded as fair-quality studies, and those that received an average score of below 2.0 were regarded as poor-quality studies. In the current review, only studies of at least fair quality (i.e., with an average quality score ≥2.0) are included in the analysis. Since seven studies had an average quality score lower than 2.0, eleven studies were included and taken to the next step in the review process, and seven studies were excluded (Table 2) [16–22].

### Extraction of data

After the study selection process, data were extracted from the selected articles. Two authors (ROC and AJvB) independently extracted the study characteristics (author, year, outcome variable(s), sample characteristics, presumed determinants, measurement instruments, and significant results (statistical figures that were reported in the article), of studies selected for inclusion. Differences were discussed and consensus was reached. In case of disagreement, a third author was contacted (CBT). Continuous rather than categorical statistics were extracted from articles when both were reported.

### Rating the levels of scientific evidence

To synthesize the results, the principles of best evidence synthesis [11, 13, 14] were applied. Specifically, information was incorporated on the number of studies, the methodological quality of the studies, and the consistency of the results. This rating system is based on levels of evidence as described by review groups from the Cochrane Collaboration [12]. Results were considered consistent (Table 3) when at least 75 % of the studies demonstrated results in the same direction, according to statistical significance of *p* < 0.05.

**Fig. 2:**
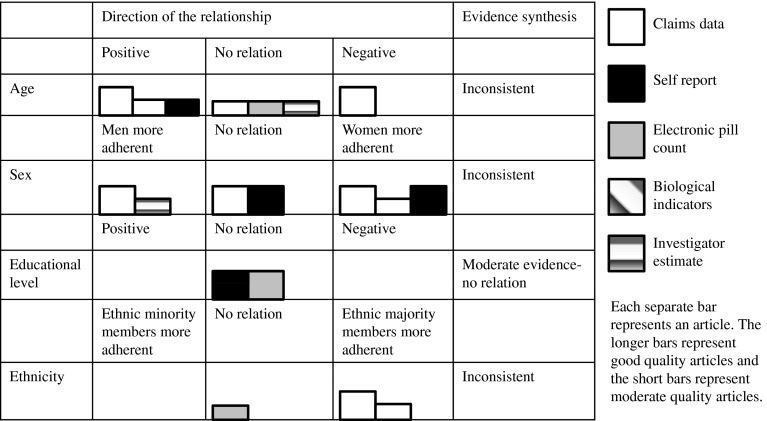
Harvest plots displaying the potential determinants found more than once in the literature, direction of the relationship found, best evidence synthesis results and techniques for measuring adherence for socio-economic factors

**Fig. 3:**
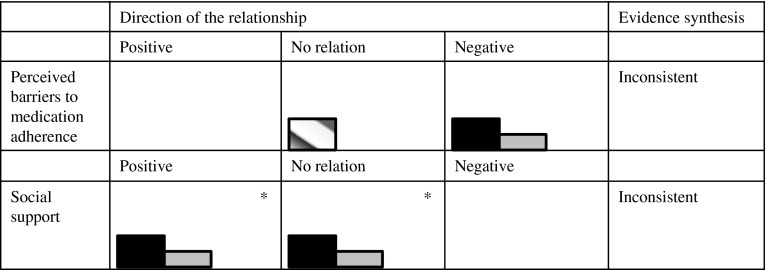
Harvest plots displaying the potential determinants found more than once in the literature, direction of the relationship found, best evidence synthesis results and techniques for measuring adherence for patient-related factors. *Social support was investigated in two studies. These studies are represented by four* bars* because there was variation within the studies on the relationship between social support and medication adherence, depending on how social support and how medication adherence were measured

**Fig. 4:**
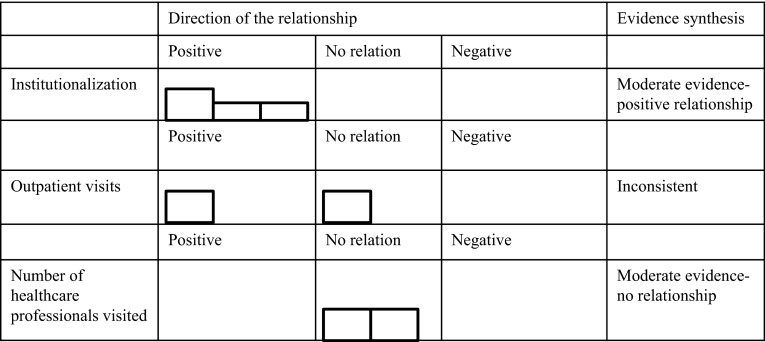
Harvest plots displaying the potential determinants found more than once in the literature, direction of the relationship found, best evidence synthesis results and techniques for measuring adherence for healthcare system-related factors

**Fig. 5:**
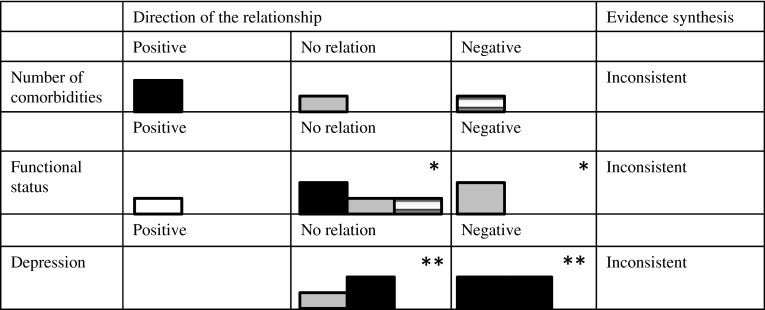
Harvest plots displaying the potential determinants found more than once in the literature, direction of the relationship found, best evidence synthesis results and techniques for measuring adherence for condition-related factors. *Funcional status was investigated in four studies, but in one [27] there was variation within the studies on the relationship between social support and medication adherence, depending on how social support and how medication adherence were measured. **Depression was investigated in three studies, but in one [26] there was variation within the studies on the relationship between depression and medication adherence, depending on how depression and how medication adherence were measured

In the results section, only the results on presumed determinants that were investigated in more than one of the included studies are discussed, since, according to the best evidence synthesis, only the evidence regarding determinants which were investigated more than once can be synthesized. However, the results that were found in single studies are displayed in Table 4. The results section is organized according to the WHO multidimensional adherence model [10]. In addition, results are depicted in so called Harvest plots [23] in order to provide a visual overview of the number and quality of the studies that showed positive relationships, negative relationships, or no relationships between the determinants and medication adherence (Figs. 2–5). Moreover, these results are stratified by measurement technique of the outcome variable.

## Results

### Studies selected

As previously described, a number of steps led to the final selection of studies included in the review. The titles and abstracts of 6683 articles were scanned and assessed for relevance, leading to a selection of 87 articles (Fig. 1). These articles’ full texts’ were scanned, and the inclusion and exclusion criteria were applied. This step led to a selection of eighteen studies eligible for inclusion, or 20.7 % of the 87 articles from the previous step. From the 69 excluded studies, 26 (37.1 %) had HF self-care behaviors other than medication adherence (including self-care management, self-care maintenance, sodium, alcohol and fluid intake restriction, physical activity, monitoring signs and symptoms, and keeping follow-up appointments) as their outcome, which were included in a previous review [15], but excluded in the current review. These studies were regarded as not meeting inclusion criterion (IC) 3, described previously. Nineteen additional studies did not meet IC 3, and therefore, 45 of 69 (65.2 %) studies in total did not meet IC 3. The other 24 of the 69 excluded studies were excluded for the following reasons: Four articles did not meet IC 1, three did not meet IC 2, and five did not meet IC 5. Moreover, two articles were excluded because they met exclusion criterion (EC) 1, three met EC 2, and seven met EC 3 (Fig. 1).

### Description and quality of the studies/data

The next step in the study selection process included an assessment of the studies’ methodological quality. This step led to an exclusion of seven of eighteen studies (38.8 %), due to being having a poor methodological quality according to the quality assessment. There were a few pervasive methodological issues. Only one [22] of the seven excluded studies (14.2 %) had a theoretical background. Only in one [18] of the excluded studies, the number of reasons for loss to follow-up was reported. In only one [21] of the excluded studies, the potential determinants were defined, while for one of the excluded studies this criterion was deemed irrelevant (16.6 %). Finally, in only one [20] of the excluded studies, the number of missing values in the data was reported.

 On the other hand, in most [24–30] included studies (seven of the nine studies for which this criterion was deemed relevant, 77.7 %), the presumed determinants were clearly defined. The outcome variable was operationalized in all included studies. Multivariate analyses were performed in all but one [26] of the included studies (90.9 %).

Six of eleven (55 %) included studies were rated good quality [24–27] and five as fair-quality studies [30–33] based on their average quality score (Table 2). The main limitation of fair-quality studies were lack of theoretical background and no reporting of missing values in the data (Table 2).

**Table 2:** Quality assessment scores

Studies generated by search, numbered by quality score	Quality criteria																	Average quality score	Quality rating
	1	2	3	4	5	6	7	8	9	10	11	12	13	14	15	16	17		
1. Roe et al. [24]	3	3	3	I	3	2	3	3	3	3	3	3	3	3	1	3	3	2.8	Good
2. Bagchi et al. [25]	1	3	I	I	I	I	3	3	I	I	3	3	3	I	1	3	3	2.6	Good
3. Cholowski et al. [26]	3	3	3	I	3	2	3	3	I	3	3	3	2	3	3	2	3	2.6	Good
4. Molloy et al. [27]	3	3	3	3	2	2	3	3	3	3	1	3	3	3	1	3	3	2.6	Good
5. Sayers et al. [28]	1	3	3	3	2	1	3	3	3	2	3	3	3	2	3	3	3	2.6	Good
6. Schweitzer et al. [29]	1	3	1	1	3	I	3	3	3	3	3	3	3	3	3	3	3	2.6	Good
7. Wu et al. [30]	3	3	1	1	1	1	3	3	3	3	3	3	3	3	1	3	3	2.4	Fair
8. Evangelista et al. [34]	1	3	3	3	1	1	1	3	3	3	1	3	3	3	1	3	3	2.3	Fair
9. Monane et al. [31]	1	1	I	I	1	1	I	3	3	I	3	3	1	3	1	3	3	2.1	Fair
10. Rodgers et al. [32]	1	3	I	I	3	2	1	1	3	I	1	3	2	3	3	3	1	2.1	Fair
11. Granger et al. [33]	1	3	1	1	3	I	I	2	2	2	1	3	2	2	1	3	3	2.0	Fair
12. Artinian et al. [22]	3	1	1	1	1	1	1	3	3	1	3	3	3	3	1	2	I	1.9	Poor
13. Evangelista et al. [16]	1	3	1	1	1	1	1	3	2	3	1	3	3	3	1	2	I	1.9	Poor
14. George and Shalansky [17]	1	3	2	2	1	1	1	1	I	3	1	3	3	3	1	3	1	1.9	Poor
15. Lamb et al. [18]	1	3	I	I	1	3	1	1	2	2	1	3	3	2	1	3	1	1.9	Poor
16. Roe et al. [21]	1	1	I	I	1	1	3	3	3	I	1	3	3	I	1	3	1	1.9	Poor
17. Pamboukian et al. [19]	1	3	I	1	1	1	I	I	1	2	3	3	1	I	1	3	3	1.8	Poor
18. Ruf et al. [20]	1	3	1	1	2	1	1	1	1	3	3	3	2	3	2	2	1	1.8	Poor

Some criteria were deemed irrelevant for some studies. These cases appear in the table as ‘I’. Studies assessed as ‘poor’ had an average quality score of less than 2 and were not included in the synthesis of the evidence

**Table 3:** Best quality synthesis applied on the extracted results

Level of evidence	Consistent findings in multiple (≥2) high-quality studies	Strong evidence
	Consistent findings in one high-quality study and at least one fair-quality study or consistent findings in multiple fair-quality studies	Moderate evidence
	Only one study available or inconsistent findings in multiple studies (≥2)	Inconsistent evidence

Seven of the included studies were conducted in the US [24, 25, 30–32], two were conducted in Australia [26], one in the UK [27], and one included samples from 25 countries [33] (Table 4). All included studies reported on different samples (32 samples in total). Different methods were used to measure adherence in the different studies, including medication possession ratio (MPR), medication event monitoring system (MEMS), interviews with patients, and questionnaires (Table 4). All included studies had demographic characteristics as presumed determinants, and in most articles [24, 25, 29, 31–34], determinants related to healthcare use, patients’ medical condition, and aspects of the prescribed medication were investigated as potential determinants.

**Table 4:** Characteristics, methods, and results of the included studies

Number, Name of Author, date, country, N, Age and Sex	Type of medication adherence and measurement tools	Determinants found significant	Results
1. Roe et al. [24] USA N = 869 Age = 60 Men = 51 %	Medication compliance, measured by medication possession ratio (MPR), continuation of therapy and dosing, were calculated based on medical and pharmacy claims data from a database containing information on more than 1.1 million Americans belonging to numerous health plans MPR: supply of ACEi/number of days between the first claim and ACEi during the post period and the end of the post period Continuation of therapy: Termination date minus the date of the index prescription Dosing: mean milligrams dispensed per day = the mg per tablet (or capsule) multiplied by the quantity of medication dispensed, divided by the days supply as indicated by pharmacist. Mean mg. dispensed per day was added across prescriptions and divided by the total number of prescriptions, leading to a mean dispensed dose per prescription. Mean percentage of an adequate daily dose dispensed was calculated as the mean milligrams dispensed per day divided by the adequate daily dose for the medication	MPR: 1. Sex (male) 2. Chronic disease score 3. Systolic proxy diagnosis 4. Outpatient visits 5. New user 6. Renal insufficiency 7. Enalapril 8. Lisinopril 9. Switched medication 10. Antihyperlipidemic agents Presumed determinants for which a non-significant (NS) relationship with medication adherence was found: 11. Prior myocardial infarction 12. Other ACE inhibiter medication Continuation of therapy: 1. Sex (male) 2. Outpatient visits 3. New user 4. Renal insufficiency 5. ACE inhibitor Enalapvil 6. Switched medication 7. Digitalis Presumed determinants for which a NS relationship with medication adherence was found: 8. Lisinopril 9. Other ACE inhibiter 10. Other cardiovascular drugs Dosing: 1. Outpatient visits 2. New user 3. Enalapril 4. Lisinopril 5. Other medication 6. Switched medication 7. Other hypertensive agents 8. Beta blockers	MPR: 1. B = 0.047, *p* < 0.05 2. B = −1.23, *p* < 0.05 3. B = 0.045, *p* < 0.05 4. B = 0.132, *p* < 0.001 5. B = −0.099, *p* < 0.0001 6. B = −0.159, *p* < 0.005 7. B = −0.072, *p* < 0.05 8. B = 0.110, *p* < 0.0005 9. B = 0.126, *p* < 0.0001 10. B = 0.048, *p* < 0.05 Continuation of therapy: 1. β = 0.56, *p* < 0.005 2. β = 0.46, *p* < 0.0001 3. β = 2.70, *p* < 0.0001 4. β = 2.16, *p* < 0.001 5. β = 1.85, *p* = 0.05 6. β = 0.25, *p* < 0.005 7. β = 0.76, *p* < 0.05 Dosing: 1. B = 0.159, *p* < 0.01 2. B = −0.196, *p* < 0.0005 3. B = 0.284, *p* < 0.0005 4. B = 0.504, *p* < 0.0001 5. B = 0.767, *p* < 0.0001 6. B = 0.463, *p* < 0.0001 7. B = 0.265, *p* < 0.0005 8. B = 0.133, *p* < 0.05
2. Bagchi et al. [25] USA N = 45572 Age = unknown Men = 28 %	MPR and persistence were used to measure adherence to therapy. Data extracted from Medicaid files MPR: the number of days a patient was supplied with more than one CHF drug in relation to the patient’s first and last prescription dates Persistence: The number of days of continuous use of CHF medications per month	Determinants of medication possession ratio: 1. Arkansas 2. Indiana 3. New Jersey 4. Age 65–74 year 5. Age 75–84 year 6. Age >85 year 7. Comorbid coronary artery disease 8. Comorbid diabetes mellitus 9. Dually eligible 10. Disabled 11. Arkansas 12. Men 13. Black race 14. Other/unknown race 15. CHF-related hospitalization in 1998 16. Non-CHF related hospitalization in 1998 17. High Chronic Disease and Disability Payment System scores 18. Percentage of generic CHF drugs Determinants of persistence: 1. Indiana 2. New Jersey 3. Arkansas 4. Age 65–74 5. Age 75–84 6. Age >85 7. Dually eligible 8. Disabled 9. Comorbid coronary artery disease 10. Comorbid diabetes mellitus 11. CHF-related hospitalization in 1998 12. Non-CHF related hospitalization in 1998 13. Black race 14. Other/unknown race 15. Non-CHF-related hospitalization 16. Chronic Disease Payment System risk score 17. Percentage of generic CHF drugs	MPR: 1. β = 1.51 (SE 0.433) 2. β = −4.79 (SE 0.422) 3. β = 1.97 (SE 0.400) 4. β = 2.14 (SE 0.489) 5. β = 4.45 (SE 0.563) 6. β = 5.27 (SE 0.644) 7. β = 5.42 (SE 0.307) 8. β = 4.75 (SE 0.310) 9. β = 1.72 (SE 0.388) 10. β = 2.58 (SE 0.411) 11. β = −4.79 (SE 0.422) 12. β = −1.19 (SE 0.314) 13. β = −6.23 (SE 0.337) 14. β = −4.94 (SE 0.385) 15. β = 2.59, (SE 0.285) 16. β = −1.65, (SE 0.289) 17. β = −2.75 (SE 0.174) 18. β = −0.06 (SE 0.004) Persistentce: 1. β = 0.55 (SE 0.130) 2. β = 0.52 (SE 0.120) 3. β = −1.08 (SE 0.127) 4. β = 0.63 (SE 0.147) 5. β = 1.24 (SE 0.169) 6. β = 1.65 (SE 0.193) 7. β = 0.45 (SE 0.116) 8. β = 0.63 (SE 0.123) 9. β = 1.26 (SE 0.092) 10. β = 1.12 (SE 0.093) 11. β = 0.90 (SE 0.086) 12. β = −0.28 (SE 0.086) 13.β = −1.50 (SE 0.101) 14. β = −1.28 (SE 0.116) 15. β = −0.28 (SE 0.087) 16. β = −0.72 (SE 0.052) 17. β = −0.02 (SE 0.001) All *p* < 0.01, reference groups for state: California
3. Cholowski et al. [26] Australia N = 54 Age = 72 Men = 61 %	Medication compliance was measured with a semi-structured interview. Four compliance behaviors were measured: forgetting to take medication, being careless about taking medication, stopping to take medication when feeling better, stopping to take medications because of feeling worse as a result of taking it	Stopping to take medications as a result of feeling worse: 1. Not complying when feeling worse as a result taking medication was related to number of co morbidities 2. Being careless about taking medication was related to depression 3. Being careless about taking medication was related to perceiving barriers to dietary compliance 4. Men were more likely to be careless about taking medications 5. Total compliance scores (including the four compliance behaviors) were related to beliefs about medication compliance (including both of the scales about perceived benefits and barriers) 6. Total compliance scores were related to the perceived barriers scale (but not the perceived benefit scale) when the scales were assessed separately Presumed determinants for which a NS relationship with medication adherence was found: 7. Number of medications 8. Number of risk factors 9. Proactive coping 10. Reflective coping 11. Strategic planning 12. Preventative coping 13. Instrumental support seeking 14. Avoidant coping 15. Self-regulation 16. Benefits to medication compliance 17. Beliefs about dietary compliance 18. Age	1. r = −0.43, *p* < 0.05 2. r = −0.31, *p* < 0.05 3. r = −0.35, *p* < 0.05 4. t = −2.16, *p* < 0.05 5. r = −0.33, *p* < 0.05 6. r = −0.42, *p* < 0.05
4. Molloy et al. [27] UK N = 147 Age = 80 Men = 57 %	ACE activity measured with serum from clotted blood	Illness beliefs about the following topics: 1. Length of the condition and the cyclical nature of it 2. The consequences of the condition 3. The personal control patients have over their condition 4. That treatments will be effective 5. That the illness makes sense 6. That it will make them emotionally distressed 7. That the illness has symptoms Presumed determinants for which a NS relationship with medication adherence was found: 8. Time-line acute/chronic	Time-line cyclical Consequences Personal control Treatment control Illness coherence Emotional representations Identity (These determinants were found to be significantly related to adherence at *p* = 0.10. We only regard significant relationships as those with a *p* value < 0.05)
5. Sayers et al. [28] USA N = 163 Age = 63 Men = 96 %	A four-item questionnaire	1. Emotional support Presumed determinants for which a NS relationship with medication adherence was found: 2. Instrumental support 3. Family involvement	β = −0.41, *p* < 0.05
6. Schweitzer et al. [29] Australia N = 115 Age = 64 Male = 71 %	The heart failure compliance questionnaire	Presumed determinants for which a NS relationship with medication adherence was found: 1. Age 2. Gender 3. NYHA 4. LVEF 5. Depression 6. Anxiety 7. Self-efficacy	
7. Wu et al. [30] USA N = 134 Age = 61 Men = 70 %	The measurement tool used was a medication monitoring system (MEMS): an unobtrusive microelectronic monitoring device in the caps of medication bottles. With this system, medication adherence was indicated with: 1. Dose count: the % of prescribed doses taken 2. Dose days: the % of days that right number of doses were taken 3. Dose time: the % of doses that were taken on schedule	Dose count: 1. Treatment-related barriers 2. Socio economic 3. Perceived social support Presumed determinants for which a NS relationship with medication adherence was found: 4. Gender 5. Age 6. Attitudes 7. Knowledge 8. NYHA 9. Comorbidity 10. Depression 11. Number of pills taken per day 12. Medication frequency 13. Patient-provider relationship 14. Educational level 15. Financial status Dose day: 1. NYHA 2. Barriers 3. Financial status 4. Perceived social support Presumed determinants for which a NS relationship with medication adherence was found: 5. Gender 6. Age 7. Attitudes 8. Knowledge 9. Comorbidity 10. Depression 11. Number of pills taken per day 12. Medication frequency 13. Patient-provider relationship 14. Ethnicity 15. Educational level 16. Financial status 17. Perceived social support Dose time: 1. Treatment-related barriers 2. Financial status Presumed determinants for which a NS relationship with medication adherence was found: 3. Gender 4. Age 5. Attitudes 6. Knowledge 7. NYHA 8. Comorbidity 9. Depression 10. Number of pills taken per day 11. Medication frequency 12. Barriers 13. Patient-provider relationship 14. Ethnicity 15. Educational level 16. Perceived social support	Dose count: 1. β = 0.352, *p* < 0.001 2. β = −0.208, *p* = 0.025 3. β = −0.241, *p* = 0.014 Dose day: 1. β = 0.181, *p* = 0.049 2. β = 0.349, *p* < 0.001 3. β = 0.208, *p* = 0.036 4. β = −0.221, *p* = 0.026 Dose time: 1. β = 0.268, *p* = .008 2. β = 0.216, *p* = .039
8. Evangelista et al. [34] USA N = 82 Age = 54 Men = 38 %	A modified version of the Compliance Questionnaire	1. Age 2. Neuroticism Presumed determinants for which a NS relationship with medication adherence was found: 3. Race 4. Education 5. Marital status 6. Mental health 7. Physical health 8. Health satisfaction	1. Adjusted R^2^ = 0.185, *p* = .000 2. Adjusted R^2^ = 0.252, *p* = .006
9. Monane et al. [31] USA N = 7247 Age = 77 Men = 21 %	Digoxin filling during 12 months: Nr. of days without therapy was computed and used as a measure of (non)compliance	Estimated number of days without therapy by: 1. Age 2. Race 3. Female gender 4. Institutionalization (hospitalization or nursing home stay) 120 days prior to digoxin prescription 5. Number of pharmacies used 120 days prior to digoxin prescription, 6. Number of non-study medications 120 days prior to digoxin prescription 7. Concurrent congestive HF medications 120 days prior to digoxin prescription Presumed determinants for which a NS relationship with medication adherence was found: 8. Age 75–84 9. Number of physicians seen	Number of days without therapy: 1. Older than 85: −17.0 days (CI −23.7, −10.3) 2. Other (not white or black): 13.6 days (7.3, 19.9) 3. −18.7 days (−24.6, −12.8) 4. −34.4 days (−39.7, 29.1) 5. 16.0 days (9.9, 22.1), 6. 4 to 7 medications −6.4 days (−12.3, −0.5) 8 or more medications 7.4 days (−13,3, −0,7) 7. Yes: −56.3 days (−61.4, 51.2) (*p* value for all <0.05)
10. Rodgers et al. [32] USA N = 64 Age = 65 Men = 57 %	Medication non-adherence was calculated as follows: Non-adherence by percent acquisition = days supply dispensed/actual days between refills × 100. It is not specified how they had data to make this calculation	1. Age 2. NYHA class 3. Hyperlipidemia 4. Asthma/COPD 5. Number of hospitalizations in the past year Presumed determinants for which a NS relationship with medication adherence was found: 6. Gender 7. Race 8. Number of years with congestive HF 9. Number of visits to primary care physician in the previous year 10. Number of health care professionals seen in previous three months 11. Visits to pharmacist managed outpatient clinics 12. Payment method 13. Number of enalapril doses per day 14. Number of other medications 15. Number of individual doses of all medications per day 16. Notation of adverse effects of enalapril 17. Tobacco or alcohol use	Predictors of non-adherence: 1. Age group 57–64 OR 17.8 Age group 65–72 OR 1.9 Age group 73–89 OR 3.3, 2. NYHA class II OR 0.04 NYHA class III OR 0.08 3. OR 0.09 4. OR 0.09 5. OR 0.16
11. Granger et al. [33] 25 participating countries (CHARM trial) N = 7599 Age = 66 Men = 78 %	Compliance was estimated by patients report, investigators’ inspection of pill bottles and tablet count in case of uncertainty	1. Gender (female) 2. Number of comorbid illnesses 3. Heart rate 4. Presence of pacemaker 5. Number of medications Presumed determinants for which a NS relationship with medication adherence was found: 6. Age 7. NYHA class 8. Ejection fraction 9. Systolic blood pressure 10. Body mass index 11. Smoking (current)	1. β = −0.049 *p* ≤ 0.001 2. B = −0.041 *p* = 0.001 3. B = −0.051 *p* = 0.000 4. B = −0.027 *p* = 0.019 5. B = 0.030 *p* = 0.022

*NS* nonsignificant

### Results of the best evidence synthesis

#### Social and economic factors

##### Age

Eight of eleven included studies measured age as a potential determinant of adherence to medication among HF patients. However, these studies did not demonstrate consistent results. Therefore, the evidence for the relationship was found to be inconsistent according to best evidence synthesis. Specifically, four of the eight studies that investigated age as a potential determinant [25, 31, 32, 34] showed a significant relationship between age and adherence, while four did not find a significant relationship between age and adherence [26, 29, 30, 33] (Fig. 2).

Of those that did find a significant relationship, three studies [25, 31, 34] found that higher age is related to more adherence. Another study [32] compared four age groups (range 57–89) and found that age group 35–56 had the highest level of adherence and age group 56–64 had the lowest level of adherence (Fig. 2).

##### Sex

Seven studies [24, 25, 29–33] investigated sex as a potential determinant of medication adherence. Due to conflicting results, the evidence for the relationship was found inconsistent according to best evidence synthesis. Although in five studies (71 %) significant relationships between sex and adherence were found, in three, it was demonstrated that men were more adherent than women [24, 31, 33], while in two, it was shown that men were less adherent than women [25, 31] (Fig. 2). In another study, it was shown that men were more likely to be non-adherent when they experienced feeling physically bad as a result of taking medication [26]. However, in the latter study, sex was not significantly related to non-adherence to medication that has made one feel better or to being careless about taking medications. Finally, in three studies, no significant relationship between sex and adherence was found [29, 30, 32] (Fig. 2).

##### Educational level

The relationship between medication adherence and educational level of HF patients [30, 34] was investigated in two studies. In both of these studies, educational level was not found to be related to medication adherence. Since both of these studies were rated as fair-quality studies (as indicated by the length of the bars in Fig. 2), according to best evidence synthesis, there is moderate evidence that patients’ educational level is not related to their level of adherence.

##### Ethnicity

Ethnic minorities were found to be less adherent than the majority ethnic groups in three [25, 30, 31] of five studies (60 %) that investigated this relationship, but in two, no relationships were found [32, 34]. Therefore, according to best evidence synthesis, the evidence for this relationship is inconsistent due to insufficient evidence (<75 %), indicating that the relationship exists. Specifically, two studies demonstrated that African Americans were less adherent then Caucasians [25, 31]. One of them also showed that people from ‘other’ races were more adherent than African Americans and Hispanic people were less likely to be adherent than Caucasians [30]. Among the studies that did not find significant relationships, one differentiated between African Americans and Caucasians [32], and the other between African Americans, Caucasians, and people of ‘other’ races [34]. As can be seen in Fig. 2, pharmacy claims data were used to measure adherence in the two studies that demonstrated that ethnic majority group members are more adherent than minority group members. However, in the study that did not demonstrate this relationship, an electronic pill device was used (Fig. 2).

#### Patient-related factors

##### Social support

Two studies [28, 30] investigated whether the level of social support that patients receive is related to their level of medication adherence. Both articles used the multidimensional Scale of Perceived Social Support to measure social support, but only one study analyzed the emotional and instrumental support subscales separately [28]. In this study, a relationship was found between medication adherence and emotional support but not instrumental support. The degree of family members’ and friends’ involvement in patients’ care was also measured in this study, using the Medical Care Questionnaire. However, a relation to medication adherence was not found for this measure of social support.

The other article [30] found that social support, including emotional and instrumental support, was significantly related to adherence when it was calculated as the amount of correct doses taken in a given day (dose day) but not when it was calculated as the correct amount of doses taken at the right time (dose time). As can be seen in Fig. 3, both studies that found a relationship used pharmacy claims data. The study that did not show a significant relationship used an electronic pill count measure.

In sum, although significant positive relationships were found between social support and medication adherence, this was not the case when instrumental support was measured independently or when the degree of family’ and friends’ involvement was used as a measure of social support. Therefore, the evidence for the relationship is inconsistent according to best evidence synthesis,

##### Patient-perceived barriers to medication adherence

The barriers to medication adherence, as perceived by patients, were measured with questionnaires in the three studies that measured it as a potential determinant of medication adherence. This relationship was found to be inconsistent according to best evidence synthesis, because these studies demonstrated conflicting evidence. Patients’ perceived barriers were found to be related to adherence in two studies [26, 30] (Fig. 3). In one [26], barriers to medication adherence were negatively related to being careless about taking medication. In this study, beliefs were measured with an adapted version of the Compliance Beliefs Scale [35]. Examples of items in this questionnaire include: “If I take my water pills, I will lower my chance of being in the hospital” and “Taking water pills is unpleasant.” In another study [30], barriers to medication adherence were related to less adherence, and this was measured with the Medication Adherence Scale [36] that includes patient-identified barriers such as “having no support from family or someone to remind me to take medications” or “confusing the mediation times.” Finally, a third study did not find significant relationships between beliefs and medication adherence [27]. This study used the Illness Perceptions Questionnaire to measure beliefs [37]. In this study, a cutoff *p* value of 0.10 was set, and the results are considered significant by the researchers.

#### Healthcare system-related factors

##### Healthcare services utilization

Four included studies [24, 25, 31, 32] investigated whether various aspects of healthcare services utilization were related to medication adherence. These aspects include institutionalization (hospitalization and nursing home stays), outpatient visits, and number of healthcare professionals visited by patients. All of these studies used pharmacy claims data to measure adherence (Fig. 4).

Moderate evidence, according to best evidence synthesis, was found for the relationship between institutionalization and adherence. This relationship was investigated in three studies [25, 31, 32], of which one was of good quality and two were of fair quality, (Fig. 4). Although evidence for the relationship was found in three studies, only one of them is of good quality. This means that according to best evidence synthesis, the evidence for this relationship is moderate. The evidence from these studies indicates that having been institutionalized in the past is related to higher levels of adherence. In another study, a variable including both hospitalization and nursing home stays was found to be related to adherence [31]. Interestingly, it was also found that hospitalization for other conditions than congestive HF was related to a lower adherence level [25].

Another aspect of healthcare services utilization, which was investigated in multiple included studies, was outpatient visits. Best evidence synthesis showed that the relation between number of outpatient visits and medication adherence in HF patients had inconsistent evidence. One study [24] found a positive association between number of outpatient visits and adherence, but another study [32] did not find a relation between number of visits to the primary care physician and adherence and between number of visits to a pharmacist-managed outpatient clinics and adherence.

In addition, number of healthcare professionals seen is yet another aspect of healthcare services utilization, which was investigated in two included studies of moderate quality (Fig. 4). According to best evidence synthesis, there was moderate evidence that seeing *more* healthcare professionals is not related to more adherence [31, 32]. One of the studies investigated this by measuring the number of physicians seen [31], while the other measured the number of healthcare professionals seen in general [32].

All of the studies that measured the relationship between the various aspects of healthcare services utilization described previously and medication adherence, used retrospective claims data as a measure of medication adherence (Fig. 4). These studies calculated medication possession ratio, continuation of therapy, persistence, percentage acquisition of drugs, or prescription fillings (specific descriptions of how these were calculated in the different studies can be seen in Table 4).

#### Condition-related factors

##### Comorbidities

Four included studies investigated whether the number or type of HF comorbidities is related to HF patients’ medication adherence. According to best evidence synthesis, the evidence for the relationship between number of comorbidities and adherence is inconsistent, due to conflicting results in the three studies that investigated it. The number of comorbidites of HF patients was significantly related to their adherence levels in two studies [26, 33] that had opposite results (Fig. 5). Namely, one study found that patients who felt worse after taking medications and had more comorbidities were less likely to stop taking medication [26]. Another study found that having more comorbidities were related to less adherence [33]. An additional study measured the relationship between adherence and a risk score of comorbidity and overall health status (using the Chronic Disease and Disability Payment System scale) and found that patients who had higher scores (more risk) had lower adherence rates [25]. In addition, one study found a nonsignificant relation between comorbidity and medication adherence [30].

Three studies measured the relationship between having a specific comorbidity and medication adherence. Having the following comorbidities was related to higher adherence levels: coronary artery disease, diabetes mellitus, hyperlipidimemia and asthma/COPD [24, 25, 32]. Only having renal insufficiency was found to be related to less adherence and only when adherence was calculated as medication possession ratio; when it was calculated by measuring the continuation of therapy, it was related to more adherence [24]. No relation was found between having a prior myocardial infarction and medication adherence [24]. It is not possible to synthesize the evidence, because each of the aforementioned comorbidities was investigated in a single study.

##### Functional status

Functional status was measured with the New York Heart Association (NYHA) class in the four studies that investigated whether it is a potential determinant of medication adherence. The evidence for the relationship, according to best evidence synthesis, was found inconsistent because the evidence in the four studies that investigated this relationship was conflicting. One study demonstrated that patients with higher NYHA had lower adherence [30] when calculating the percentage of days that the correct doses were taken (Fig. 5). However, this study did not find a significant relationship between NYHA class and adherence when calculating adherence as the percentage of doses taken or the percentage of doses taken on schedule. Another study demonstrated that patients with higher NYHA had lower *non*-adherence [32], and three studies [29, 30, 33] found a nonsignificant relationship between these two variables.

##### Depression

The evidence from the three studies that investigated the relationship between depression and medication adherence was inconsistent. Two studies [29, 30] demonstrated a nonsignificant relationship (Fig. 5). In another study [26], it was found that depressed patients were more likely than non-depressed patients to be careless about taking medication but were not more likely than non-depressed patients to stop taking medication when the medication made them feel unwell, or when they were feeling better. Depression was measured with questionnaires in these studies: the Patient Health Questionnaire in one study [30] and with the Beck Depression inventory in two others [26, 29]. In two of the three studies, adherence was measured by self–report, while in the other study, it was measured by an electronic pill device (see Fig. 5).

#### Treatment-related factors

##### Aspects of the prescribed medication

Six of eleven studies (54.5 %) measured potential determinants regarding the prescribed medication (including being prescribed various types of medications, having switched from one type of medication to another, number of medications prescribed, frequency of having to take medication, and treatment-related barriers). Of these, in three studies [25, 31, 33], significant relationships were found between aspects of the prescribed medication and adherence (50 %); in two studies [24, 30], a significant relationship was found with some aspects of the prescribed medication but not with other aspects (33.3 %), and in one study [32], a relationship was not found (16.6 %). The specific aspects of the prescribed medication are displayed in the results table (Table 4). It is not possible to synthesize the findings of these results in order to draw conclusions, because each of these studies measured different aspects of the prescribed medication. However, since in five of eight studies that investigated this relationship (62.5 %) some significant relationships were found, there is an indication that aspects related to the type of medication prescribed may be related to adherence rates.

## Discussion

The current review is the first to systematically assess the evidence regarding presumed determinants of medication adherence and to employ a quality assessment and a best evidence synthesis. It is the most rigorous review on determinants of medication adherence among HF patients to date.

In the current review, eleven studies were included, six of which were regarded as fair- and five as good-quality studies. The reviewed articles reported on relationships between medication adherence and a wide range of potential determinants. However, most of the relationships were rated inconsistent, usually because of conflicting evidence for the relationships between the different determinants. One relationship had moderate evidence, namely the relationship between adherence and institutionalization. In addition, there was moderate evidence that educational level and seeing more healthcare professionals is not related to medication adherence.

The results regarding institutionalization are difficult to interpret. On the one hand, having had an institutionalization (including hospitalization and nursing home stays) in the past (the exact timeframe varied between the different studies) was found to be related to higher levels of adherence in the current review. On the other hand, the relation between number of outpatient visits and adherence was found to be inconsistent. Moreover, non-HF-related hospitalizations were found to be unrelated to adherence in one study [25]. There may be some differences between these types of healthcare services that explain this result. It could be that during hospitalization patients are informed and even educated about the medications they should take, that as the nurses pass by to give patients medications they also stress the importance of taking medication and that this does not occur during outpatient visits. It could also be that more evidence is needed about the effects of outpatient visits, which would change the picture. Finally, number of healthcare professionals seen was found to be unrelated to medication adherence, which means that seeing more professionals does not improve adherence, but does not harm it either.

Another possible explanation is that patients that have worse health, and therefore have higher institutionalization rates, are more motivated to adhere in order to reduce their symptoms. However, the evidence for the relationship between functional status and adherence was rated inconsistent in the current review due to conflicting evidence. It could also be that patients’ *perceptions* of their health drive motivation to adhere to treatment. It is possible that during hospitalization patients develop a more negative view on their health leading them to adhere to their medications after discharge from the hospital.

Finally, it is possible that patients who have experienced hospitalization become scared about being readmitted to the hospital. One study [38] gives an indication for this interpretation. In this study, the adherence rates to self-care recommendations of a group of patients receiving an educational in- and out-hospital intervention were compared with those of a control group receiving usual care. The results showed that patients in both groups increased their adherence levels after discharge, but that patients who were in the intervention group sustained this improvement for a period longer than 1 month. This shows that recently discharged patients may be more likely to adhere to recommendations. More research is needed to establish why institutionalization benefits adherence and which types of institutionalization benefit it. Although institutionalization is not recommended as an intervention to increase patients’ adherence, perhaps providing adequate education to institutionalized patients could increase adherence.

It is important to keep in mind that all the results regarding healthcare services utilization in general, and institutionalization in particular, come from pharmacy claims data. These data demonstrate the rates at which medications were claimed from pharmacies, but do not demonstrate the rates at which medications were consumed by patients. Therefore, these results should be interpreted with caution. It remains to be seen whether patients that were institutionalized in the past and are more likely to claim medications are also more likely to consume the medications they have claimed.

Although ethnicity had inconsistent evidence, this may be due to the measurement techniques used to measure adherence. In the two studies that found that ethnic majority group members are more adherent than minority group members, pharmacy claims data were used. In the one study that did not find an association between ethnicity and adherence, electronic pill counting device was used to assess adherence. This means that it could be the case that ethnic majority group members claim more medication than ethnic minority group members, but they do not consume more medication. In addition, the findings regarding relationships between ethnic groups and medication adherence may be mediated by socio-economic status. More research is warranted to clarify this topic.

Almost all studies that measured the relation between a specific comorbidity and adherence found that patients who had comorbidity were more adherent than those who did not. However, studies that assessed the relation between number of comorbidities and medication adherence paint an inconsistent picture. It could be that it is not the *number* of comorbidities that makes a difference, but rather the type. Again, more research is needed in order to establish the nature of the relationship.

It was apparent in the current review that aspects related to the medication could be relevant determinants of adherence. Perhaps different medications pose different barriers to patients, because of different experienced side effects. However, since each study included in the current review measured a different aspect of the medication prescribed, it is unclear which aspects are the important ones. More research is warranted.

One of the aims of the current work is to provide recommendations for future interventions. This aim could not be met due to the lack of consistent results on potential modifiable determinants of medication adherence among HF patients. It is suggested that future studies on HF patients’ medication adherence investigate modifiable determinants that have been found to affect levels of adherence in other populations than HF. Important psychological variables, such as self-efficacy, perceived benefits and barriers and perceived risks of medication have only been investigated in three of the included studies [26, 27, 29] but are constructs that could potentially be targeted in interventions that aim to increase adherence to medication. More research is warranted on these as well as other potential determinants that could be targeted in interventions.

The current work reveals that few studies investigating presumed determinants of medication adherence among HF patients are available to date. The studies that are available use a variety of methods to investigate both presumed determinants and adherence levels, which makes it difficult to compare the results. Although it has been suggested that a diverse variety of variables affect medication adherence of older adults [39], the studies available to date on medication adherence among HF patients demonstrate that many potentially important determinants have only been investigated in a limited number of studies at best. Some examples of possibly important determinants include factors related to patients’ ability to acquire and retain information, such as cognitive decline and health literacy. Variables such as patients’ living conditions and ability to purchase medication may also be relevant. Such variables are not found in the literature.

The evidence was conflicting and therefore regarded inconsistent according to best evidence synthesis for most potential determinants that were investigated more than once. There are a few reasons for this. It could be that different studies used different measurement instruments to measure adherence, which may have led to variability in the results. Another reason could be that different sub-samples of HF were included in different studies, such as patients of different age groups, different geographical areas, and different levels of HF severity, and that for each of these sub-groups different determinants are important.

The fact that there was a scarcity of studies of at least fair quality limits the ability to draw far-reaching conclusions and is a limitation of the current work. We reviewed eleven studies and regarded only five of these as good-quality studies. Notably, in the current review, seven of eighteen studies were excluded from the analysis because they were regarded as poor-quality studies. The main quality issues (as can be seen in Table 2) that these studies had were related to lack of theoretical framework (all excluded studies received the lowest possible score on this quality item), not reporting whether the study had enough participants available for analysis and not having provided definitions of potential determinants and covariates (4 of 5 studies received the lowest possible score on these items). It is especially important that future studies are of high methodological quality.

Based on our results, it becomes apparent that there is no clear profile for non-adherent HF patients, so it is not possible to point to specific types of patients to whom interventions to increase adherence should be directed. However, it is apparent that institutionalization may benefit HF medication adherence, possibly because having a patient education program centered only on HF is beneficial to adherence. Therefore, a possible intervention to increase adherence rates may be education on HF medications during hospitalization. More research is needed on other determinants of HF medication adherence to allow making more recommendations for future interventions, since most of the available evidence is conflicting.
